# Horizontal Gene Transfer Involving Chloroplasts

**DOI:** 10.3390/ijms22094484

**Published:** 2021-04-25

**Authors:** Ewa Filip, Lidia Skuza

**Affiliations:** 1 Institute of Biology, University of Szczecin, 13 Wąska, 71-415 Szczecin, Poland; lidia.skuza@usz.edu.pl; 2 The Centre for Molecular Biology and Biotechnology, University of Szczecin, 13 Wąska, 71-415 Szczecin, Poland

**Keywords:** plants, cpDNA, HGT, chloroplast genes, endophytes

## Abstract

Horizontal gene transfer (HGT)- is defined as the acquisition of genetic material from another organism. However, recent findings indicate a possible role of HGT in the acquisition of traits with adaptive significance, suggesting that HGT is an important driving force in the evolution of eukaryotes as well as prokaryotes. It has been noted that, in eukaryotes, HGT is more prevalent than originally thought. Mitochondria and chloroplasts lost a large number of genes after their respective endosymbiotic events occurred. Even after this major content loss, organelle genomes still continue to lose their own genes. Many of these are subsequently acquired by intracellular gene transfer from the original plastid. The aim of our review was to elucidate the role of chloroplasts in the transfer of genes. This review also explores gene transfer involving mitochondrial and nuclear genomes, though recent studies indicate that chloroplast genomes are far more active in HGT as compared to these other two DNA-containing cellular compartments.

## 1. Introduction

Horizontal gene transfer (HGT), also called lateral gene transfer (LGT), is the process of transferring genetic material between organisms by routes other than parent-offspring (vertical gene transfer—VGT). HGT can involve the transfer of genetic material from one cell to another: between different bacterial species by processes of conjugation, transduction, and transformation; between different single-celled organisms, e.g., bacteria and yeasts; or between even more distantly related organisms, e.g., bacteria and insects, fungi and animals, or plants and fungi. In 1984, Syvanen introduced and gradually developed the concept of HGT as interspecies gene flow [1,2]. The phenomenon was first discovered in 1951 in Klebs-Löffler bacillus (*Corynebacterium diphtheriae*). It was observed that the gene responsible for pathogenicity, *tox*, was of viral origin and could transfer from pathogenic to non-pathogenic bacteria. In 1959, it was shown that bacterial genes responsible for antibiotic resistance could also be transferred via this route. HGT has further been shown to have a significant role in the evolution of eukaryotes. Above all, the role of this process in protists is to be highlighted. HGT can be considered a universal phenomenon observed in the genomes of bacteria, fungi, plants, or animals [1,2]. HGT is commonplace in prokaryotes [3,4] as demonstrated by studies typing approximately 81% of genes in which transfer was noted [5]. In recent years, the readability of eukaryotic genomes in next generation sequencing (NGS) studies has facilitated the characterization of the occurrence and mechanism of HGT in eukaryotes [6].

Moreover, data on the entire genomes of prokaryotes revealed the presence of HGT between distant species [7,8]. Extensive research on the participation of HGT in prokaryotic evolution (e.g., archaea and bacteria) has shown a possible mechanism to facilitate the acquisition of new features [9]. However, this phenomenon has been speculated to be rare in eukaryotes [10,11].

Transmission and integration of transferred genes can provide several beneficial features, including prokaryotic adaptation during environmental changes [12,13], acquisition of new features/functionalities [14], and evolutionary adaptation in eukaryotes [15,16]. Most cases revealed one transfer of one gene in one species, but recent findings reported a case of massive transfer of at least tens, and probably hundreds, of foreign mitochondrial genes in *Amborella trichopoda*.

**Figure 1:**
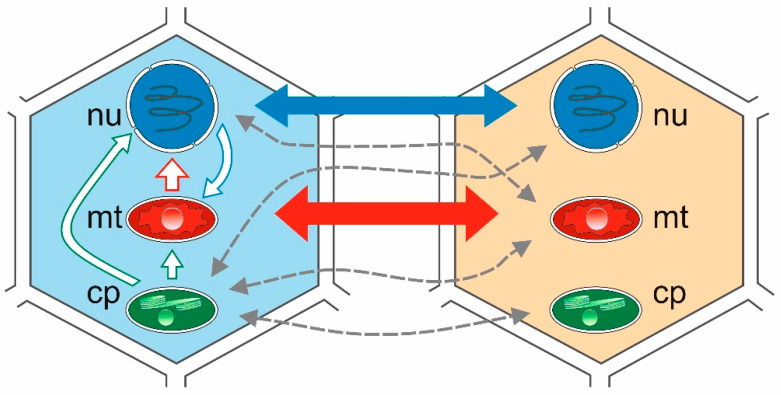
Intracellular gene transfer (IGT) and horizontal gene transfer (HGT) between two plant species. Intergenomic gene transfer represent lines in the cell from the left. Solid blue, green, and red lines depict the number of reported HGT events between cell compartments. Thicker lines indicate more frequent events than thinner lines. Dashed grey lines depict putative transfers. cp: chloroplast; mt: mitochondria; nu: nucleus.

There are also other mechanisms, e.g., intracellular transfer involving cell organelles, such as the nucleus, the mitochondria, and the chloroplasts (intracellular gene transfer—IGT) [17], or the transfer of genetic material to a different location in the genome of the same organism (intragenomic transfer or transposition) (Figure 1). Given the endosymbiotic origin of mitochondria and chloroplasts, many genes of eubacterial origin migrated from these organelles to the nucleus via IGT as well as horizontal transfer. Many recorded transfers occurred relatively recently during evolutionary history and were limited to a single recipient, or to a small number of species within the genus. However, while the IGT rate has dropped significantly since the appearance of eukaryotes, it remains a common process characteristic of the evolution of the nuclear and organelle genomes in plants [18,19,20,21,22,23]. Among the three types of genomes in a plant cell, there are six possible directions for gene transfer. The most prominent are from the organelle (mitochondrial) genome to the nuclear genome [22,23], and from the nuclear and plastid (chloroplast) genome to the mitochondrial genome [18]. Intracellular transfer to the highly compact plastid genome appears to be quite rare; however, it is being reported increasingly frequently. More information can be found in the literature indicating numerous cases of nuclear-to-mitochondrial IGT transfer than from chloroplast to mitochondrion or to cell nucleus [18].

Recent studies have shown that plant mitochondrial genomes are more likely to transfer DNA to the other two cellular compartments mentioned above. The ever-increasing number of plant mitochondrial genomes (mtDNA) sequenced [20,24,25,26,27] reveals the extent of transfer from both chloroplast (cpDNA) and nuclear genomes (nDNA) [28,29,30,31,32,33,34,35,36,37,38]. In general, plant mtDNAs consist of between 0.56% (*Marchantia polymorpha*) and 10.85% (*Phoenix dactylifera*) plastid-derived sequences [33]. The integration of nuclear sequences is usually more complex and more difficult to identify, as it involves retrotransposons and other repetitive sequences. Repetitive sequences are common to mtDNA of various seed-bearing plants, including *Oryza sativa* [33], *Arabidopsis thaliana* [35], *Cucumis melo* [34], and *Cucumis sativus* [28], as well as *Gossypium* species [31,32]. Long terminal repeat retrotransposons (LTR-retro) typically constitute the largest proportion of plant nuclear repeats, and often have a dominant influence on nuclear and mitochondrial genome size [38,39,40,41,42,43]. A 2019 study [18] on four cotton species (*Gossypium raimondii*, *Gossypium arboreum*, *Gossypium hirsutum*, and *Gossypium barbadense*) indicated the presence of multiple nuclear and chloroplast tRNA gene repeats in the plant mtDNA genome. The authors highlighted the discovery of eight chloroplast-derived tRNA genes—*trnD*, *trnH*, *trnM*, *trnN*, *trnP*, *trnS*, *trnV*, and *trnW*—in the mitochondrial genomes of all four cotton species [18]. In this paper, we discuss mainly cases of HGT and IGT gene transfer involving the chloroplast genome of plants.

## 2. Organisms between Which HGT Occurs

In contrast to gene transfer by interspecies crossover, which is restricted to closely related organisms, there appear to be no clear taxonomic boundaries for HGT. As described above, transfers between different species are known, e.g., between bacteria and fungi, bacteria and plants, bacteria and animals, fungi and animals, and fungi and plants. The highest number of HGT cases among Eukaryotes was observed in unicellular organisms [44,45] (Table A1). The number of transferred genes in these organisms ranges from one to several dozen, comprising less than 1% of the total genome. Most were found to have come from bacterial donors. In the plant world, a small number of HGT exchanges have been observed. One of the first described was the exchange of DNA between cells of *Agrobacterium* and the cells of higher plants [46]. Additional putative cases include the acquisition of aquaglyceroporins from eubacteria (1200 million years ago) [47] and glutathione biosynthesis genes from α-proteobacteria [21].

Viral DNA does not typically integrate into the host’s nuclear genome; this has occurred only sporadically throughout evolutionary history. Random recombination between the viral genome and the plant chromosomal DNA is believed to be the molecular mechanism underlying such events [44]. 

It is worth noting that chloroplast and mitochondrial RNA polymerases encoded by the nuclear genome are similar to bacteriophage T7 polymerase, which may have contributed to the emergence of HGT transfers during the evolution of eukaryotes [40]. In addition to genes or their fragments, mobile genetic elements can also be transferred. Roulin et al. [48] reported the transfer of retrotransposon LTR Route66 between *Sorghum* and several *Oryza* species, remaining active after transfer.

Fungus-to-plant HGT (FP-HGT) plays a significant role in shaping plant genomes. Published literature on this mechanism has deepened our understanding of the genetic evolution of disease resistance, and has opened up a new avenue for the identification of plant resistance genes. It seems, therefore, that there are no genetic barriers “prohibiting” the transfer of genetic material between even evolutionarily distant organisms. Further discussion will focus primarily on plants [49,50].

## 3. HGT Routes and Mechanisms 

The mechanisms responsible for HGT are not sufficiently understood. Transfer of nucleic acids via intermediaries, such as viruses, bacteria, fungi, and transposons, or via the direct uptake of nucleic acids (especially in parasite-host systems) are usually indicated. Genetic material could theoretically be transferred by DNA fragments or by mRNA, which would then be converted back into DNA by reverse transcription. Research tends to point to the former possibility.

Two modes of transfer have been identified—vector and direct [50]. The vector route requires the presence of vectors such as bacteria, fungi, viruses, etc. [51,52], while the direct route can occur through direct DNA exchange [18]. HGT between nuclear and organelle genomes [53] has received particular attention in the literature [54]. HGT involving bacteria or fungi has also been documented [54,55]. Extensive studies suggest the possibility of HGT from plants to other genomes via pathogens, transgenic bacteria (e.g., *Agrobacterium tumefaciens*), viruses, fungi, and nematodes [56,57,58,59].

Furthermore, plant cells are characterized by the presence of three types of the genome: nuclear, mitochondrial, and chloroplast, which differ in their susceptibility to HGT. It is believed that there is a low level of horizontal transfer of nuclear genes, despite evidence of numerous horizontal movements of their genetic elements [36,60]. Some authors conclude that the nuclear genome arose through HGT by the fusion of archaebacterial and eubacterial genomes [18,60]. Given the endosymbiotic origin of mitochondria and chloroplasts, many genes of eubacterial origin have migrated from these organelles to the nucleus by intracellular modes of HGT [18,61,62,63,64] as well as by intercellular modes.

## 4. HGT and Endophytes

Recent scientific reports indicate that plant growth under natural conditions is closely linked to the microorganisms accompanying them, known as endophytes—organisms that develop asymptomatically in plant tissues for at least part of their life cycle. These can be either fungi or bacteria, including actinomycetes. Thus far, they have been isolated from numerous species of cultivated, forest, and aquatic plants [44,65]. Endophytes influence inhabited plants in various ways, which can be characterized as direct, indirect, or ecological.

Direct influences are related to the synthesis of compounds by the endophyte that destroy pathogens. Such compounds include terpenoids, alkaloids, aromatic compounds, and also lytic enzymes capable of degrading chitin, proteins, cellulose, hemicellulose, and DNA. Indirect endophyte influences on plant resistance are often related to the induction of plant defense mechanisms, such as the synthesis of secondary metabolites. Another mechanism of indirect influence is the improvement of growth and overall condition, making the host more resistant to stresses. Thus, the relationships between endophyte and plant are economically important due to their potential applications in agriculture, industry, and health, as well as environmental protection.

**Figure 2:**
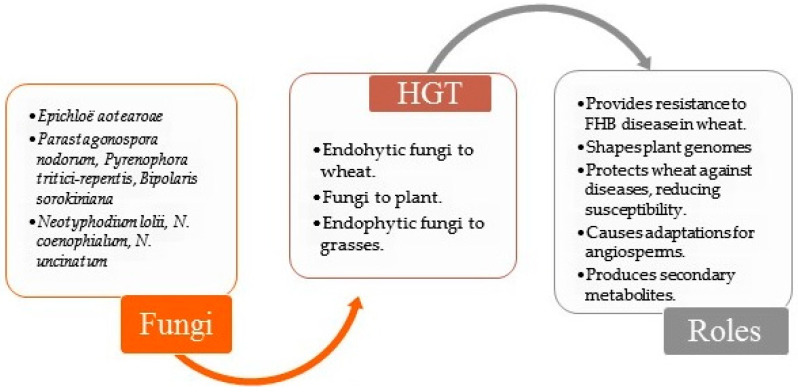
Examples of HGT cases described in plant and fungi endophyte relationships [43,66,67,68,69,70,71,72,73,74,75]. FHB—Fusarium head blight.

The phenomenon of HGT in endophytes highlights an important biological mechanism for their evolutionary adaptation within the host plant, as they continuously acquire ‘new traits’ of adaptation. It should be noted that this research problem remains unexplored in the literature. There is evidence for the role of endophytic bacteria in the biodegradation of toluene and the reduction of disease in durum wheat (*Triticum durum*) and maize (*Zea mays*) [45,49]. Furthermore, researchers have hypothesized that genetic recombination between plants and endophytes may have led to the incorporation of metabolic pathway genes into the host plant [45]. Figure 2 illustrates occurrences of transfer between plant and endophyte (bacterium or fungus).

The effects of HGT following a relationship with an endophytic microorganism represent an important process by which adaptation problems may be solved. Attention has been drawn to both the beneficial and detrimental effects of the coexistence of grasses with endophytes. Since few studies are available on this topic, we wish to highlight the importance of HGT in plants in beneficial relationships with endophytes.

Common wheat (*Triticum aestivum* L.) is a major source of calories for the human population [43,69]. The occurrence and spread of the devastating disease Fusarium head blight (FHB), exacerbated by recent climate change and certain cropping practices, poses a threat to global wheat production and food security. FHB is caused by fungi belonging to the genus Fusarium, and its occurrence is observed in all major cereals grown worldwide (wheat, triticale, rye, oats, barley, maize). FHB has the greatest impact on wheat crops. For example, *F. graminea rumist* is the main pathogen causing FHB of wheat in China, the United States, Canada, European countries, and many other countries [66,70]. This is due to the high susceptibility of this cereal, the prevalence of the pathogen, and the large area under wheat cultivation. *Fusarium* produces epoxy-sesquiterpene compounds known as trichothecenes. These compounds are protein synthesis inhibitors and virulent pathogens [70]. Trichothecene contamination of cereal grains results in immunotoxicity and cytotoxicity in humans and animals. Wheat resistance FHB is a quantitative trait [72,73,74,75], and numerous quantitative trait *loci* (QTL) associated with resistance to this disease have been reported.

In their study, Wang et al. [69] indicated that *Fhb7* (encoding glutathione S-transferase—GST) shows similar effects on FHB resistance and confers broad resistance to *Fusarium* to species. At the same time, studies of wheat genomes have been undertaken in which the *Fhb7* reference gene for *Th. elongatum* has been developed. It was found that *Fhb7* can detoxify trichothecenes by catalyzing the conjugation of a glutathione (GSH) unit onto their toxic epoxide moiety, where the active sulfhydryl group neutralizes the toxicity. In the authors’ view, the coding sequence of *Fhb7* has no obvious homology with any known sequence across the plant kingdom, but shows 97% sequence identity with a species of endophytic fungus (*Epichloë aotearoae*) known to infect grasses. This provides evidence that *Fhb7* in *Th. elongatum* probably originates from the fungus, and arrived via HGT. Furthermore, such transfer carried out experimentally has been shown to provide wheat with resistance to FHB [69,70,71].

The analysis of HGT from fungi to plants uses sequenced endophyte genomes to understand how fungal pathogens interact with plants. For some time now, it has been possible to find information in the literature about the *ToxA* gene that is produced by two fungal pathogens of wheat, *Parastagonospora nodorum* and *Pyrenophora tritici-repentis*. It has been hypothesized that these pathogens shared *ToxA* and part of the surrounding repetitive DNA via HGT, resulting in a sequence of 11,000 bases that is almost identical between the two species [76].

Subsequent genome sequencing results indicated the presence of *ToxA* in the genome of *Bipolaris sorokiniana* infecting wheat and barley. Interestingly, the *ToxA* identification in the pathogen genome has an identical 11,000 kbp fragment. The high identity between these three species of *Parastagonospora nodorum*, *Pyrenophora tritici-repentis*, and *Bipolaris sorokiniana* shows that HGT must have occurred very recently [77,78]. *Parastagonospora nodorum* and *Pyrenophora tritici-repentis* are believed to share *ToxA* and a number of DNA sequences, presumably through HGT, resulting in an 11 kbp transfer which is almost identical between *Parastagonospora nodorum* and *Pyrenophora tritici-repentis*. These fungi are the main causes of diseases in cereals such as root rot, leaf spot disease, seedling blight, and black point. The high homology between these three pathogens indicates further evidence of HGT.

This also highlights the importance of *ToxA* itself, which plays a significant role in wheat diseases. *ToxA* is responsible for necrosis (cell death) in wheat leaves during infection. It does this in a very specific way, acting in a gene-for-gene relationship with the gene responsible for the susceptibility of wheat to fungal diseases: *Tsn1*. If both genes are present, *ToxA* in the fungus and *Tsn1* in wheat, the infected leaf dies. If either gene is absent, there are no *ToxA*-related symptoms. This has been confirmed by studies showing that when the pathogen is a vector, *ToxA* has a significant effect on disease symptoms in wheat varieties containing the *Tsn1* gene. Thus, *ToxA* plays a key role in the disease caused by these pathogens. Work on this phenomenon further solidifies HGT as a mechanism by which fungal pathogens can share strategies to exploit host vulnerabilities. HGT is, of course, not limited to bacterial plant pathogens. In the case of *ToxA*, it has been shown to weaken wheat resistance to various fungal diseases. All of these diseases occur worldwide in wheat crops, meaning that *ToxA* poses a global threat to the yields of common wheat [77,78].

## 5. Intracellular Gene Transfer

As described above, DNA fragments can migrate from the cell nucleus of one organism to the cell nucleus of another organism. This process can also occur between all the elements of a cell containing genetic material: nucleus, mitochondria, and plastids. The mitochondria of most seed-bearing plants examined have both nuclear and plastid sequences. Mitochondrial genes are also found in plastids, but rarely. The difference is probably due to the fact that mitochondria, unlike plastids, have efficient mechanisms to take up foreign DNA. Many genes of mitochondrial origin have been found in nuclei. In such cases, prokaryotic genes are converted into eukaryotic genes, which are related, among other things, to the fact that they undergo recombination during sexual reproduction. Presumably, RNA is involved as a mediator in this type of IGT [18,19,20,21,22].

### 5.1. HGT in Cell Nuclei and Plastids

Many traces of HGT have also been found in the nuclei of angiosperms. These concern nuclear genes as well as transposons. One interesting case is the parasitic plant *Rafflesia cattleyi*, in which more than 30 genes have been found that have been transferred from the host. At least some of these are functional. Plastids, on the other hand, are thought to be highly resistant to incoming processes of HGT or IGT. Plastid sequences, rather, are found in other genomes of the cell—mitochondrial and nuclear—having transferred from a starting point in the plastid [79,80,81].

### 5.2. Chloroplast-to-Mitochondria Transfer

Today, HGT into mitochondria occurring between distantly related higher plant species is a well-known phenomenon, and is no longer as controversial as when the work by Woloszynska et al. was released [82], which was the first to demonstrate horizontal transfer of DNA sequences from chloroplasts to mitochondria. The subject of the study was a fragment of chloroplast *trnA* gene intron, named *pvs-trnA* because it is part of the *pvs* sequence that determines male sterility. The *pvs-trnA* sequence was identified in only three species of the genus *Phaseolus* and it was found that, although it contains only 190 bp, it differs from the chloroplast *trnA* sequence of beans in as many as 10 positions and is most similar to the chloroplast genes of *Philodendron scandens* and *Magnolia grandiflora*, showing only three differences. In view of this, the phylogenetic trees generated placed *pvs-trnA* between plants from the class of monocotyledons and the order of magnoliales, at a position isolated from the leguminous plants to which beans belong. The results of this study demonstrated that the *pvs-trnA* sequence did not arise from intracellular transfer from chloroplasts to mitochondria of the same plant, but from horizontal transfer of a *trnA* intron fragment from the chloroplasts of a non-dicotyledons plant to mitochondria of a plant of the genus *Phaseolus* [82].

Among the cases of horizontal gene transfer from plant to plant that have been studied, in about 1 in 40 cases, a gene of mitochondrial origin encoding respiration-associated proteins or ribosomal proteins had been transferred. To date, only one exception has been described: the horizontal transfer of a fragment of the intron *pvs-trnA* described above. Subsequently, in *Isoetes engelmannii*, an insert of chloroplast origin containing *trnA* and 23S rRNA gene sequences was identified in the mtDNA. These sequences are highly similar to their counterparts in the cpDNA of *I. malinverniana*, indicating inter-organelle transfer rather than HGT [83].

Recent data indicate that between 1.1% and 6.3% of mitochondrial DNA has been transferred via the IGT pathway from chloroplasts [27]. Chloroplast sequences are more often transferred to the mitochondrial genome, and also by pathways other than HGT, e.g., via IGT [84,85,86,87,88,89,90,91,92,93,94,95,96], which is why we find traces of chloroplast-derived sequences in the mtDNA of genera such as *Arabidopsis*, *Beta*, *Brassica*, and *Oryza* [87]. This is not surprising since the homology of certain chloroplast and mitochondrial genome sequences had already been noted in the 1980s [89]. According to Hao and Palmer [89], the mitochondrial genome of flowering plants tends to adopt chloroplast sequences that have no effect on its function [92] as these were progressively degraded to pseudogenes.

It was, therefore, decided to look for homologous recombination (and gene conversion) events between the sequences of the two genomes. Consequently, it was possible to observe a repeated conversion—the replacement of short mitochondrial sequences of the *atp1* gene by chloroplast homologous *atp1* sequences. Both homologues encode an α subunit of ATP synthase. It is likely that, after integration of the *atp1* gene into the mitochondrial genome, intra-mitochondrial recombination occurred. The chimeric *atp1* genes in the mitochondrion are presumed to be functional, to have ORFs and to not exhibit the characteristics of pseudogenes, and it is likely that substitutions in the recombinant region are synonymous [89]. Analysis of HGT cases in plants has also led to the introduction of the term “duplicative” HGT, which is linked to conversion. Duplicative HGT involves the transfer and integration of foreign genes (single genes, their fragments, whole assemblies) which, in the recipient mitochondrial genome, do not immediately replace their counterparts. HGT, together with recombination, contributes to the genetic diversity of mtDNA [90].

### 5.3. Mitochondrion-to-Chloroplast Transfer of Rps16

An interesting case is the transfer of the *rps16* gene, which is present in the chloroplast genome of most higher plants, while it is absent in the cpDNA of *Medicago truncatula* and *Populus alba*. In these species, the *rps16* gene is present in the mitochondrial genome. The product of mitochondrial gene expression may be directed to both mitochondria and chloroplasts. Such targeting of the mitochondrial product of the *rps16* gene is also characteristic of *Arabidopsis thaliana*, *Lycopersicon esculentum*, and *Oryza sativa*, in which the gene is present in both organellar genomes (Table 1) [94].

### 5.4. Chloroplast-to-Nucleus Transfer

The structure and gene content of the plastid genome are well conserved among different land plant species [101]. The chloroplast genome of land plants has a highly conserved organization. It is a circularly shaped, double-stranded DNA molecule that contains two inverted repeat (IR) copies that separate a small and a large single-copy region (small single copy—SSC; large single copy—LSC). The cpDNA contains genes that encode proteins responsible for the mechanisms of photosynthesis, and biosynthesis of fatty acids, amino acids, pigment, and vitamins, among others [102]. The region of inverted repeats usually contains three rRNA genes (*rns*, *rnl*, *rrn5*) and two tRNAs (*trnA*, *trnI*), but there may be more as a result of contraction or by the expansion of this region. Nevertheless, the gene repeats are rarely identical. Plastids are characterized by the presence of proteins that are encoded by nuclear genes and post-traumatically imported into their region. Moreover, genes in the plastid genome show a high degree of similarity among a wide variety of plants. Therefore, it is believed that most genes were transferred from the original endosymbiont to the nucleus at an early stage of plant evolution [103,104]. However, evidence for the ongoing occurrence of chloroplast gene transfer to the nucleus has been demonstrated. It is believed that the *tufA* gene, encoding the chloroplast elongation factor *Tu*, was transferred from the chloroplast to the nucleus in the algae line [105,106].

During research on sequencing of the tobacco chloroplast genome, a homologue of the Escherichia coli *rp122* gene, encoding the ribosomal protein L22, was found [95]. This gene was subsequently found in the cpDNA of monocotyledons and dicotyledons, vascular land plants, and in algae [96,97]. However, *rp122*, which is part of a large region of ribosomal protein genes in rice, tobacco, and liverwort, is not found in its normal position in the cpDNA of soybean and subterranean clover (*Trifolium subterraneum*) [95]. Thus, it can be concluded that the plastid genomes of legumes do not contain *rp122*, which raises the question of whether this gene has been completely lost from legumes or has been transferred to the nucleus. There is evidence that in pea, *rp122* is located in the cell nucleus, which has been confirmed by detailed studies involving the structure of this gene and clarifying its transfer mechanism [95]. Studies on this case, initiated in the 20th century, showed that sequences corresponding to the exon and intron of pea *rp122* had not been found in any characterized chloroplast gene. Thus, long-term studies on the evolutionary transfer of pea *rp122* have completely placed it outside the angiosperm chloroplast gene clade. This demonstrates that this gene was transferred to the nucleus long before its subsequent loss from the chloroplast genome of its legume ancestor. Gantt et al. [95], based on the best phylogenetic tree they have obtained in the course of their study, showed that nuclear transfer probably preceded the loss of chloroplasts by at least 100 million years.

A 2001 publication [96] showed that among the six fully sequenced chloroplast genomes of angiosperm plants (with the exception of the non-photosynthesizing plant *Epifagus virginiana*), most protein-coding genes are universal. In contrast, other genes are species-specific, e.g., *accD*, *ycf1*, and *ycf2* (pseudogenes in rice and maize), *rpl23* (pseudogene in spinach), and *infA* (pseudogene in tobacco, *Arabidopsis*, and *Oenothera elata*) [106]. Chloroplast genes have been lost in angiosperms, including *rpl22*, *rps16*, and *ycf4* (*ORF 184*), which have been lost in some or all of the legumes [96]. Furthermore, *ycf2* and *ndhF* were repeatedly lost in various angiosperm species [96]. Another example of the loss of the chloroplast genes, i.e., *infA*, *rps16*, *ycf1*, *ycf2*, and *ycf4*, has not been elucidated, as it is not known whether they reflect successful gene transfer to the nucleus or complete loss of the gene from the cell. A generally better-understood case is *rpl22* in legumes, as it represents a classic chloroplast-to-nucleus gene transfer where the protein is imported back into the chloroplast via a transit peptide [96]. In the case of analysis of the chloroplast locus *rpl23* in spinach, it was shown to be a pseudogene and has been functionally replaced with a nuclear gene similar to the homologous cytosolic ribosomal protein gene.

Further confirming the case for DNA sequence transfer is the chloroplast *infA* gene, which has been repeatedly lost in the evolution of angiosperms. Studies have shown that the four nuclear *infA* genes characterized so far probably arose by independent gene transfer processes from the chloroplast to the nucleus. The changes in *infA* in angiosperm cpDNA have been shown to be similar to the evolution of *rps10* in mitochondrial DNA of angiosperms [96], as evidenced by loss and for nuclear transfer in both cases (Table 1) [94,97,98,99,100,101,102,103,104,105,106,107].

## 6. Conclusions

In conclusion, it is important to note the importance of HGT in plant evolution. Currently, extensive literature shows that HGT plays a powerful role in eukaryotes. Remnants of this process are found in all major groups of organisms. For example, the important role that transfer has played in plant evolution has been demonstrated by the transformation of the intracellular prokaryotic endosymbiont into a chloroplast. A very interesting form of evolution occurs when pathogens exchange DNA between species, which, as described above, is a biological process known as horizontal gene transfer (HGT). The HGT events that have been observed by detecting genes or DNA sequences which are almost identical between distantly related species are very interesting. In this paper, we have only highlighted a few of the many such cases where a single horizontally transferred gene can contribute to the acquisition of many important traits for plants. In particular, we have highlighted cases of fungus-to-plant transfer that confer adaptive plasticity in plants to new and extreme conditions, efficient stress responses, more efficient DNA repair, and the ability to neutralize toxicity.

Still, little is known about the functional and implications of HGT between plants, other than the confirmed presence of nuclear and mitochondrial HGT in the plant kingdom. This phenomenon raises a number of questions regarding the scope and mechanism of the exchange of genetic material between plants. There is a lack of information on the consequences for the storage of a given transgene and the flow of information between the organelle (cpDNA, mtDNA) and the nuclear genome. It should be emphasized that few studies indicate cases of sequence transfer of chloroplast origin. Current advances in generating genomic comparative data will contribute to the rapid discovery of evolutionary aspects of HGT between plants, including confirmation of the involvement of cpDNA.

## Figures and Tables

**Table 1:** Plastid genes in HGT involved in HGT in plants and type of the transfer.

Gen(s)	Transfer Type	Ref.
*rpl22*	chloroplast to nucleus	[95]
*infA*	chloroplast to nucleus	[96]
*tufA*	chloroplast to nucleus	[95]
*rpl32, rps122*	chloroplast to nucleus	[91,92,93,94,95,96,97,98,99,100]
*pvs-trnA*	chloroplast to mitochondrion	[83]
*rps16*	chloroplast to mitochondrion	[94]
*rps16*	mitochondrion to mitochondrion	[94]

## Abbreviations

HGT	Horizontal Gene Transfer
LGT	Lateral Gene Transfer
VGT	Vertical Gene Transfer
IGT	Intracellular Gene Transfer
IGT	Intragenomic Gene Transfer
cpDNA	chloroplast genome
nDNA	nuclear genome
mtDNA	mitochondrial genome
LTR	LTR-retro retrotransposons
FP-HGT	fungus-to-plant HGT
FHB	Fusarium head blight
IR	inverted repeat cpDNA
SSC	small single copy cpDNA
LSC	large single copy cpDNA

## Appendix A

**Table A1:** Selected examples of organisms in which horizontal gene transfer was found.

Gene Donor	Recipient of Gene	References
**Bacteria:**	**Plant:**	[108]
*Agrobacterium rhizogenes*	*Nicotiana*	
**Plant:**	**Plant:**	[109]
*Oryza*	*Zea mays*	
**Plant:**	**Plant:**	[110]
Flowering plant:	*Gnetum*	
*Asterid*		
**Plant:**	**Plant:**	[58]
*Monocot*	*Aktinidia*	
*Eudicot*	*Amborella*	
*Unknown*	*Betulaceae*	
*Ranunculales*	*Caprifoliaceae*	
*Monocot*	*Sanguinaria*	
**Bacteria:**	**Fern:**	[111]
*Nostoc azollae*	genus *Azolla*	
**Plant:**	**Plant:**	[112]
Multiple grass lineages	*Alloteropsis semialata*	
**Cyanobacteria:**		[113]
*Calothrix*	**Alga:**	
**Alga:**	*Euglena myxocylindracea*	
*Porphyra purpurea*		
**Bacteria:**	**Yeast:**	[114]
*Lactococcus* sp.	*Saccharomyces cerevisiae*	
**Plant:**	**Plant:**	[115]
Flowering plants	*Amborella trichopoda*	
Mosses		
**Plant:**	**Plant:**	[82]
*Eudicot*	*Phaseolus vulgaris*	
**Plant:**	**Plant:**	[116]
*Fabales*	*Apodanthaceae*	
**Plant:**	**Plant:**	[117]
*Orobanchaceae*	*Plantago*	
**Bacteria:**	**Yeast:**	[118]
*Pseudomonas* spp.	*Saccharomyces cerevisiae*	
**Plant:**	**Plant:**	[119]
Flowering plant:	Fern:	
*Santalales*	*Botrychium virginianum*	
**Plant:**	**Plant:**	[120]
*Ericaceae*	*Ternstroemia*	
*Cyrillaceae*		
**Plant:**	**Plant:**	[121]
Flowering plant:	Flowering plant:	
*Populus*	*Populus*	
**Cyanobacteria:**	**Alga:**	[122]
*Crocosphaera watsonii*	*Heterocapsa triquetra*	
*Trichodesmium erythraeum*	*Karlodinium micrum*	
	*Oxyrrhis marina*	
**Plant:**	**Plant:**	[123]
*Poa palustris*	*Festuca ovina*	
**Plant:**	**Plant:**	[124]
*Orobanche*	*Phelipanche*	
**Fungus:**	**Plant:**	[125]
*Physcomitrella patens*	*Oryza sativa*	
*Coprinopsis cinerea*	*Arabidopsis thaliana*	
*Laccaria bicolor*		
**Plant:**	**Plant:**	[126]
*Cuscuta*	*Plantago*	
**Dinoflagellate:**	**Dinoflagellate:**	[127]
***Prorocentrum minimum***	*Prorocentrum minimum* nucleus	
**Alga**	**Fern:**	[128]
**Bacteria**	*Mankyua chejuense Helminthostachys zeylanica Botrychium ternatum*	
**Bacteria**	**Alga:**	[129]
	Ochrophytes	
**Bacteria:**	**Nematode:**	[130]
*Wolbachia*	*Onchocerca volvulus*	
**Bacteria:**	**Animal:**	[131]
***Wolbachia***	Insect:	
	*Callosobruchus*	
**Bacteria:**	**Animal:**	[132]
*Wolbachia*	Insect:	
	*Drosophila ananassae*	
**Alga:**	**Animal:**	[133]
*Vaucheria litorea*	Snail:	
	*Elysia chlorotica*	
**Bacteria:**	**Animal:**	[134]
*Wolbachia*	Insect:	
	*Aedes aegypti*	
**Cyanobacteria:**	**Protozoan:**	[135]
*Synechococcus* sp. *Prochlo rococcus* sp.	Amoeba:	
	*Paulinella chromatophora*	
**Human:**	**Protozoan:**	[136]
*Homo sapiens*	Malaria parasite:	
	*Plasmodium vivax*	
